# Dynamics on global brain networks at the neuronal resolution

**DOI:** 10.1186/1471-2202-16-S1-P128

**Published:** 2015-12-18

**Authors:** Masanori Shimono, Ryota Kobayashi

**Affiliations:** 1 Department of Physics, University of Indiana, Bloomington, IN, 47405, USA; 2 Harvard/MGH, 149 13th St, Charlestown, MA, 02129, USA; 3 National Institute of Informatics, 2-1-2 Hitotsubashi, Chiyoda, Tokyo 101-0003, Japan

## 

A key question in computational neuroscience is the way in which microscopic components work together within the macroscopic brain scale. We demonstrate a computational model simulating the whole brain activity gathering neuronal components through columnar architectures.

We used a Multi-timescale adaptive threshold (MAT) model for the single neuron model, which has proven to be one of the most accurate models for reproducing the spike trains of a variety of cortical neurons in vitro [1]. The parameters used for expressing excitatory and inhibitory neurons were adopted from it [2].

Before connecting through multiple brain regions, we tuned parameters about background inputs and balance of synaptic intensities between inhibitory cells and excitatory cells on local neuronal circuits [3,4]. The background inputs were designed to represent both of driving inputs from the Poisson neurons in subcortical nuclei and sustaining activities within individual cortical regions.

A past study demonstrated computational simulation of the whole mammalian brain including the thalamus by constructing a network organization of the cortex using Diffusion Tensor Imaging (DTI) [5]. We used a connectivity matrix provided from invasive tracing technique to sustain the accuracy, and especially the directionality of connectivity matrix [6,7]. Furthermore, the cortical network used in our study includes "weight" of connections [8]. We show that we can reconstruct the number of neurons from the "weight" of connections, and can design connections between neurons crossing different brain regions. The number of neurons at each brain regions allowed us to integrate whole-brain network and neuronal dynamics included in each brain region [7]. Computational modeling using non-invasive brain images is also important to use in cases of human brain structure.

As summary, this computational model demonstrates a whole brain dynamics at the resolution of the neuron level for the purpose of illuminating what parameter will be potentially critical to change the dynamics of the brain. From this computational simulation, we will show a basis of understanding how optimally the brain structure is designed from the generated dynamics, including robustness against damages on the brain.
